# Leveraging smart glasses for telemedicine to improve primary healthcare services and referrals in a remote rural district, Kingandu, DRC, 2019–2020

**DOI:** 10.1080/16549716.2021.2004729

**Published:** 2021-12-10

**Authors:** Jules Diaka, Wim Van Damme, Felipe Sere, Lenka Benova, Willem van de Put, Steven Serneels

**Affiliations:** a Memisa, Kikwit, The Democratic Republic of the Congo; b Department of Public Health, Institute of Tropical Medicine, Antwerp, Belgium; c Memisa, Brussels, Belgium; d Chair and Co-founder Iristick, Antwerp, Belgium

**Keywords:** Health system, sub-Saharan Africa, digital health, referral, rural health

## Abstract

**Background:**

Telemedicine enables new forms of medical consultation and is expanding worldwide. Patients in sub-Saharan Africa could potentially benefit substantially from telemedicine.

**Objective:**

To improve primary healthcare services, especially referrals to the district hospital, for the population in three health centres in the rural district Kingandu in the Democratic Republic of the Congo (DRC) by introducing Smart Glasses, and leveraging them for telemedicine.

**Methods:**

The project involved the design and introduction of an intervention combining community engagement with technological innovation (Smart Glasses, communication equipment, moto-ambulances, and new diagnostic tests), and with staff training. Utilisation of the intervention, use of the health centres, and referrals to the hospital were monitored through the routine health information system and project-specific registers. Key stakeholders were interviewed and the project costs were analysed.

**Results:**

The use cases for the intervention were defined in consultation with the stakeholders. Smart Glasses were used in 10% of consultations in the health centres mostly for advice during curative consultations. The total number of consultations increased significantly in the intervention health centres. The number of referrals to the hospital remained stable, but an increased proportion effectively arrived in the hospital. The Smart Glasses and moto-ambulance greatly facilitated emergency referrals, often requiring a potentially life-saving intervention in the hospital. All stakeholders involved highly valued the intervention.

**Conclusion:**

Telemedicine can contribute to improving primary healthcare services in a remote rural area, as part of a more comprehensive intervention and with intensive participation of all stakeholders. It can increase acceptability and use of the existing services; improve diagnosis, treatment, and referral of patients; and can also facilitate on-the-job training and supportive supervision.

## Background

Thanks to advances in technology, there are many new possibilities in healthcare beyond the ‘traditional’ face-to-face consultation between a patient and a caregiver. These new possibilities are commonly referred to as telemedicine, and it is expanding rapidly across the world [1,2].

There are many forms of telemedicine, such as (1) a patient can consult a provider remotely while having visual contact and allowing for a view of lesions, (2) during a face-to-face consultation between a patient and a primary care provider, a specialist can join remotely for advice and joint decision-making, or (3) for telediagnosis, e.g. assessing the blood pressure, glycaemia, or heart rhythm of patients. Telemedicine also allows for a flexible integrating of these different modes of interaction, limiting the need for transport and physical referral of patients. Such integrated systems not only allow for remote expert advice on diagnosis or treatment but also allow for training and supervision of generalist practitioners by specialists.

COVID-19-related travel restrictions and the need for physical distancing between healthcare providers and their patients have accelerated the necessity and willingness to explore the potential of telemedicine [3]. This has resulted in an accelerated adoption of telemedicine by patients and healthcare providers and triggered the rapid expansion of the availability of technology [4].

In the United States, it is estimated that consumer adoption of telemedicine has increased, from 11% of consumers using any form of telehealth in 2019 to 46% in 2020 [5]. Indeed, in many settings telemedicine was rapidly accepted as a way to triage patients to assess whether in-person visits were necessary and as an alternative for cancelled face-to-face consultations.

Telemedicine innovations hold many promises, with improved convenience and easier access to care, better patient outcomes, and a more efficient healthcare system. But often concerns are raised about the quality of care (especially about the inability to conduct physical examinations and diagnostic tests) and about equity (poorest, illiterate, marginalised populations are at risk of being excluded). Obviously, in a sensitive area like medical care, there are also concerns about ethics and privacy, with the need to protect patient rights and adhere to professional standards when using telemedicine. Therefore, it is necessary that regulatory frameworks are enacted and policies are developed by policymakers in collaboration with patients and providers as the main stakeholders. In many countries, the COVID-19 crisis has triggered rapid, and often quite drastic, changes in the legal and reimbursement policies [6] to overcome structural barriers, often seen as the most important brakes on the adoption of telemedicine by providers. Providers have rapidly scaled up telemedicine platforms, and it is estimated that remote patient contacts have increased 50- to 175-fold over the last year [5].

In sub-Saharan Africa, the region with the greatest medical need, radio communications have been used within the health system for many decades. They are progressively being replaced by mobile phones where access is possible. It is worth exploring the potential of telemedicine for improved access to quality healthcare in this region also, despite obvious limitations of communication technology and power supply. The Democratic Republic of the Congo (DRC) is a case in point, with poor health indicators, and in a context of a chronic complex humanitarian crisis with millions of people having little access to quality essential healthcare. Healthcare workers are heavily concentrated in urban areas, with few skilled staff in rural health facilities, and a generally under-funded public health system.

Over the past decades, the potential of telemedicine has been explored in various countries in sub-Saharan Africa [7]. Experiences have been reported in diverse fields, such as community health [8], mental health [7], and perinatal care [9]. Such pilot experiences are most often supported by international donors, such as the Japan International Cooperation Agency (JICA) in Ghana and Zambia [10], or implemented by international non-governmental organisations (NGOs). But, in general, telemedicine over the internet remains at an early stage in Africa [11,12], despite the important unmet healthcare needs.

Over the past years, international donors including USAID [13], DFID [14], and Grand Challenges Canada – Creating Hope in Conflict [15,16], have been supporting innovative pilots in telemedicine.

This paper reports a pilot experience to improve medical care for patients in a remote rural area in the DRC, through an intervention including telemedicine. The project introduced Smart Glasses (Iristick) [17] to facilitate communication between healthcare workers in rural health centres and hospital staff, with the double aim to improve quality of care provision in the health centres, and improve the appropriateness and outcomes of referrals from health centres to the referral hospital.

## Methods

### Setting

The intervention took place in Kingandu district, a remote rural area in the DRC. Smart Glasses and a moto-ambulance system were introduced to the public health system, composed of rural health centres and a referral hospital. Health centres are staffed by nurses and midwives providing basic primary healthcare (PHC) with a strong focus on Maternal and Child Health (MCH) and care for infectious diseases. More difficult cases need a referral to the district hospital; however, due to limited road and transport infrastructure, referrals are difficult and expensive, and patients often do not arrive, or only after important delays. Malaria is by far the most frequent diagnosis, followed by respiratory infections, and diarrhoea. There are also many private clinics, mostly informal, often staffed by unqualified providers. Public services receive limited financial resources and little supplies from the government or international donors. Only a few of the staff receive regular salaries. All services have to be paid for by the users to allow for some staff income and replenishment of drug stocks. Memisa, a Belgian NGO, provided funding for investment and training and supports the overall management of the health district.

### Aim of the intervention

The project aimed at improving the quality of services by enhancing the collaboration between health centres and the hospital. The intervention consisted of a multi-pronged strategy to improve the functioning of three health centres within the district health system by introducing in the health centres: (1) Smart Glasses to establish reliable communication to the district hospital, (2) moto-ambulances to facilitate referrals, and (3) some point-of-care tests to upgrade the diagnostic capacity of the health centre.

The telemedicine solution thus used Smart Glasses (Iristick) to unlock and leverage the scarce medical expertise available in the district hospital in Kingandu to improve medical care for the population living in the catchment area of three rural health centres.

It was anticipated that the intervention would improve referrals from the health centres to the district hospital; in particular, to increase successful needed referrals (for which hospital services, such as emergency surgery, are required) and decrease unnecessary referrals (for which hospital stay has no benefit for the patient, as compared to care at the health centre).

### Specifics of the intervention

Iristick, a start-up company that has developed and is producing the Smart Glasses obtained a grant of Canadian Dollars (CAD) 250,000 from Grand Challenges Canada and partnered with: (1) Memisa, a Belgian NGO that has been supporting the Kingandu district for many years, as implementer, (2) the health authorities of the Kingandu district, (3) Avanti, a satellite communication provider, and (4) the Institute of Tropical Medicine, Antwerp [18–20].

The four partner institutions conducted the project from April 2019 to December 2020.

The Iristick Smart Glasses are equipped with cameras, speakers, and microphones, and after initiating the call on the smartphone, the system is completely hands-free and the health centre nurse can continue the consultation as usual while getting remote ‘over-the-shoulder’ expert advice from the hospital doctor. As the Smart Glasses are designed as an extension of a smartphone, the nurse can always use all the different features of the smartphone such as calling, WhatsApp, and immediate registration of consultation-related data.

**Figure 1.**
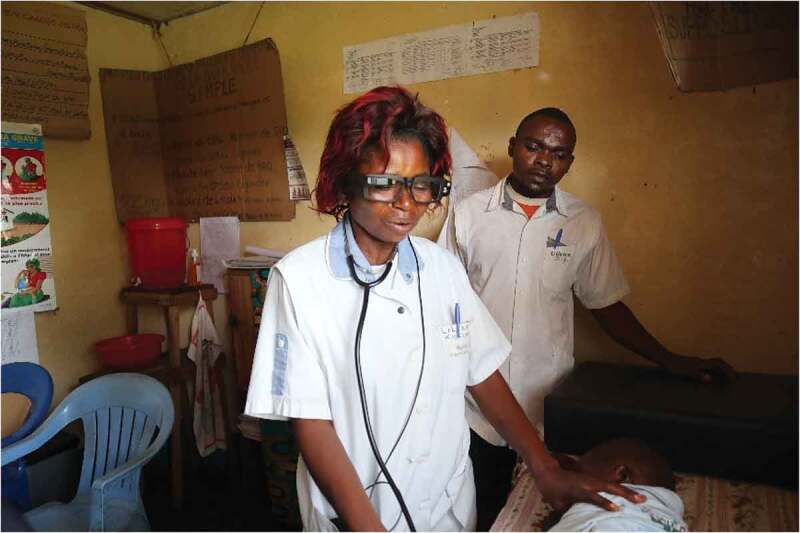
A health centre nurse wearing the Smart Glasses during a consultation

The Smart Glasses-based telemedicine system is set up as follows:

- During a consultation in a health centre, the nurse decides whether remote medical assistance is required; if so, he/she contacts the hospital via WhatsApp, addressed to all the doctors in the referral hospital, who receive that message on their smartphone.
- One of the doctors takes the call on a laptop computer dedicated for telemedicine at the hospital, while the health centre nurse continues the consultation.
- The health centre nurse wears the Smart Glasses connected to a smartphone and communicates with the doctor (Figure 1). The nurse can also see what is being streamed on the ‘heads-up display’ (small LCD screen) of the Smart Glasses.
- The doctor behind the laptop assists in the remote consultation, seeing on the screen exactly what the nurse sees through the Smart Glasses, and communicates directly to both the nurse and the patient. The doctor can decide to call other hospital staff, such as a senior midwife or laboratory technician to participate in the session, under his/her supervision.
- The doctor discusses the course of action with the nurse, specifically which patients can be treated and followed up in the health centre, and which patients need to be referred.
- If an emergency referral is needed, transport is arranged by moto-ambulance.

As there was no reliable mobile phone connection in the project area, broadband communication via satellite was installed via a solar-powered Avanti VSAT. In the district hospital and each of the three rural health centres involved in the pilot project an ‘ECO’ terminal was installed providing satellite broadband service (data speeds of 20 Mbps download and a minimum of 2 Mbps upload), using a 1.2 m antenna, 4 Ka-band satellite, and a local Wi-Fi transmitter providing a local Wi-Fi hotspot and indoor Wi-Fi Access Point. The system uses solar power cells on the roof of the health centre to power the VSAT and Wi-Fi systems.

To facilitate the transport of patients needing hospital care, a moto-ambulance system was part of the intervention, covering the routes linking Kingandu’s district hospital with the three health centres participating in the intervention, which are not always accessible by car. The moto-ambulance consists of an off-road motorcycle equipped with an additional chair (trailer or sidecar) for the patient and is stationed at the district hospital. The health centres can call the moto-ambulance when needed.

Initially, all consultations were performed with the routinely available diagnostic tools and medicines, including rapid diagnostic tests (RDTs) for malaria and HIV and a basic set of essential medicines. In a second phase, some additional laboratory resources were introduced in the three health centres: Hemocue HB 301 with MicroCuvette for assessing haemoglobin concentration [21], glucometer for assessing the level of glycaemia, and finger-tip pulse oximeter for assessing oxygen saturation.

The intervention was introduced in three rural health centres: Sondji, Kimbimbi, and Katenda, respectively at 9, 6, and 28 km from the hospital, with an estimated population of 20,500 in the coverage areas.

The implementation of the Smart Glasses pilot project was done in three phases: (1) a preparation period of five months, (2) an installation trial and training period of 12 months, and (3) a full implementation period of four months.

Phase 1: Over the period April to August 2019, the project team was set up in Kingandu and prepared the project. This involved defining the precise scope and focus of the project, selecting the pilot sites, identifying and ordering the necessary equipment, consulting the different stakeholders about the project, and obtaining ethical approval.

Phase 2: Between September 2019 and August 2020, the project staff organised the training of all staff involved in the use of the smartphones and Smart Glasses, installed the communication equipment (VSAT) and the solar panels, set up the processes for their safe use, and maintenance, including for calling and operating the moto-ambulances, and for use of new diagnostic tests. The system was only intermittently available during this period enabling several trial periods.

Much attention was given to introducing the intervention to the community stakeholders and local authorities, and involving them in the setup of the processes. This community involvement was crucial to design the moto-ambulance system, which requires the financial participation of the community for the running costs. Also, it was decided to introduce written informed consent forms for patients receiving care. The project was initially designed to improve maternal and neonatal healthcare, especially to improve emergency care during childbirth complications. During the engagement with the community and the health staff, however, it became clear that they did not favour such specific focus and insisted on a broader application of the Smart Glasses to improve all healthcare services provided in the health centres.

Consequently, three potential types of uses of Smart Glasses were identified: (1) unscheduled calls for priority and emergency advice, (2) scheduled calls during specific weekly time slots for each health centre for non-urgent advice, and (3) calls for online training and trouble-shooting between hospital and health centre staff, not only to support the introduction of the new diagnostic tools (Hemocue, glucometer, and pulse oximeter) but also for the appropriate use of RDTs and partographs.

For the newly introduced diagnostic tools, initial distance training of health centre staff was provided by the hospital laboratory. First, the laboratory experts were wearing the Smart Glasses, and the health centre staff were following on their smartphones. Thereafter, the situation was reversed with health centre staff wearing the Smart Glasses while performing tests, with a laboratory technician at the district hospital following on the computer screen.

Initially, there were numerous technical problems compounded by the lack of digital and technical literacy of the health centre staff, resulting in the frequent breakdown of the VSAT communication, especially due to lightning strikes and instability of the antennae due to strong winds. These problems were progressively solved, but the solar equipment did not allow for continuous 24-hour energy supply, with daily interruptions of connectivity during night-time hours.

Phase 3: From September to December 2020, all processes and equipment were continuously functional and used in all sites. Project staff was still needed frequently for troubleshooting and ongoing training.

### Data sources and evaluation design

We used data from the routine Health Information System of the Ministry of Health (DHIS2; national portal: www.snisrdc.com) to extract data on the utilisation of the three participating health centres.

In addition, for the project, added registers were introduced to record every use of the Smart Glasses, specifying the reason, every use of the moto-ambulance, and all referrals. All activities were monitored monthly through the DHIS 2 and the added registers.

Towards the end of the project, in December 2020, a participatory evaluation was organised to assess the perception of the intervention by the local stakeholders. In December 2020, an officer of Memisa, not involved in the project, interviewed 39 key stakeholders in the district about the project: health centre staff (10), hospital staff (5), community leaders (11), patients (11), and staff of the health district office (2). Semi-structured interviews were taped and transcribed. A thematic analysis was carried out by two researchers (WVD and WvdP) following the themes covered in the interviews: process of introduction of the innovation, and impact on their work and on the service to the patients. Early 2021, all relevant data were reviewed, triangulating the different data sources, reconstructing a project timeline, combined with insights from the thematic analysis of the interviews. We also analysed the project costs from the bookkeeping of Memisa, and the project reporting to Grand Challenges Canada.

The project was approved by the Ethics Committee of the School of Public Health of the University of Kinshasa. In line with the demand of the Ethics Committee, no images or videos from the Smart Glasses consultations were stored; they were automatically deleted after streaming. Written consent was taken from each patient before using the technology.

## Results

### Use of the smart glasses

On average, the Smart Glasses were used roughly twice a day at each clinic. The hospital staff had to manage five calls a day and approximately 39% of the calls resulted in referrals to the hospital. Over the entire project period, Smart Glasses were used 622 times during health centre consultations, for telemedicine, excluding contacts for training. In 79% of cases, this was for advice by hospital-based staff with diagnosis and treatment for curative consultations (Table 1). To a lesser degree, the Smart Glasses were used for assistance with prenatal or postnatal consultations (6%), and least frequently for advice during childbirth and newborn care (2%). In 13% of uses, this was for assistance with laboratory tests.

**Table 1.** Utilisation of the smart glasses (September 2019 to December 2020)

Purpose of use of Smart Glasses	Times used	(%)
Curative Care		
Under-5	225	
>5 years	265	
Total	490	(79%)
Pre- and postnatal care		
Prenatal	35	
Postnatal	3	
Total	38	(6%)
Delivery care		
Childbirth	7	
Newborn care	5	
Total	12	(2%)
Assistance with laboratory tests	82	(13%)
Grand Total	**622**	**(100%)**

During the initial months of introduction and fine-tuning of the intervention, a significant increase in the number of outpatient consultations occurred in the three participating health centres. This was probably mainly due to the initial enthusiasm and curiosity triggered by the activities to inform the population about the project. After a few months, the number of consultations stabilised, but remained significantly higher (+80%), than prior to the start of the project. The total number of referrals from the participating health centres to the district hospital remained relatively stable and thus decreased proportionally from 13% to 7% of all consultations. The big drop was mainly on referrals resulting from ordinary (= non telemedicine) consultation: from 9% to 3%, while the referrals resulting from telemedicine consultations remained stable at 4% of the visits.

**Figure 2.**
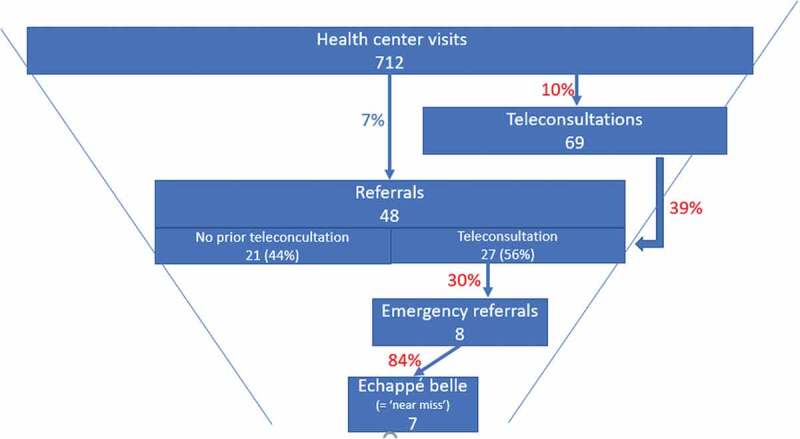
Average monthly consultations and referrals for the 3 rural health centres with smart glasses (September–December 2020)

We conducted a more detailed analysis of all the referrals of patients by the three health centres to the district hospital over the Phase 3 period September–December 2020, including analyses of the patient file of the emergency referrals to the hospital. (Table 2 and Figure 2).

**Table 2.** Average monthly consultations and referrals for the 3 rural health centres with smart glasses (September–December 2020)

	Visits without use of Smart Glasses	Visits with use of Smart Glasses	Total visits
Health centre visits	643	69	712
Referrals from the health centre to the hospital	21	27	48
Referrals arrived at the hospital	18	26	44
(Potentially) life-saving intervention during hospitalisation	0	7	7

There were on average a total of 712 monthly health centre visits, of which 48 (7%) were referred to the hospital, and 44 of these arrived (92%). That 92% of referrals arrived is a significant increase compared to the estimated 70 to 75% prior to the project. Eight of these referrals were considered emergencies, and seven of these indeed resulted in a major potentially life-saving intervention, such as a caesarean section or emergency blood transfusion of a very anaemic child; considered ‘near miss’ (or ‘échapée belle’ in French) (Table 2).

The Smart Glasses were used in 69 cases (10% of the total consultations), after which in 27 cases (39%) a referral was decided; 26 of which (96%) arrived at the hospital. Over the same period, 21 referrals were made without the use of the Smart Glasses of which 18 (86%) arrived at the hospital.

### Cost of the intervention

The total costs and investment for the project over 20 months are summarised in Table 3.

**Table 3.** Costs of the project

Category	Purpose	Cost (CAD)
Investments in equipment	Smart Glasses (including phone & software): CAD 2,650/set (special not-for-profit price)*15 for the entire project (3 health centres and district hospital, including laboratory)	39,750
	VSATs for 4 sites (special not-for-profit price)	8,000
	Motorcycles: moto-ambulance (CAD 5,000) and transport for project manager (CAD 4,000)	9,000
	Diagnostic equipment: Hemocue, glucometer, and oximeter: CAD 4,425 per site (3 sites)	13,275
Project costs including training	Project staff + travel + admin + indirect costs	163,975
	Communication (special not-for-profit price)	16,000
	**TOTAL**	**250,000**

It is estimated that recurrent costs for the continued operation of this telemedicine intervention would cost CAD 400 per month per site with VSAT, CAD 50 per month for the moto-ambulance, and CAD 300 for continued supervision and support. For the current network including the hospital and three health centres, this would add up to CAD 1,550 per month, increasing to CAD 4,700 per month for running costs of a network with 10 health centres and two moto-ambulances. The critical component of the recurrent cost is the telecommunication fees by satellite (CAD 400 per month per site). This would be reduced to only some CAD 50 per month per site, in an area with 3G network coverage.

### Perceptions by stakeholders expressed during the evaluation

All stakeholders, without exception, reported being very positive about the changes introduced. All were enthusiastic and proud about the innovative technology introduced in their community and judged that quality of care had improved thanks to the intervention.

They consistently reported that patients assessed the experience as very positive, resulting in a steep increase in the utilisation of health facilities as proof of increased trust in the healthcare system.

Community leaders and patients especially valued the direct contact with a hospital doctor, and the resulting improved trust in the decisions of the nurse, especially when this involved transfer to the hospital.

Health centre staff said they felt empowered and gained trust, and reported greater appreciation by the community and a significant increase in health centre income from user fees. Also, the additional diagnostic tests introduced allowed them to make better decisions, especially whether referral to the hospital was warranted.

Hospital staff reported improved referrals, with necessary referrals arriving more rapidly, and unnecessary referrals being avoided, especially in cases where the hospital doctor could advise on the use of appropriate treatment available in the health centre. They experienced telemedicine consultations as a useful form of direct supervision, at moments when it was most needed and directly beneficial for patients.

They also valued much that new diagnostic tools could be introduced as on-the-job training without the need for in-person workshops at the hospital, avoiding costly trips and the absence of health centre staff from their place of work, allowing for effective integration of the new diagnostic tools in routine practice.

The most important results of the introduction of the telemedicine innovation, plus moto-ambulances, as reported by the stakeholders, are summarised in Table 4.

**Table 4.** Results of the introduction of the telemedicine innovation plus moto-ambulances as reported by stakeholders

Improved diagnosis by health centre staff, thanks to support by the remote doctor in real-time, and new diagnostic tests Improved medical skills of health centre staff Improved referral system, with more timely referrals for patients needing hospital care, and avoidance of unnecessary referrals for patients that could be treated at the health centre Major improvement in the relation between the health district and the health centres. Efficient introduction of new diagnostics tests in health centres Remote formative supervision possible Improved convenience and access to care for the underserved population Increased confidence of population resulting in more people visiting the health centres and increased income from user fees

## Discussion

The project managed to operationalize a telemedicine system, in an environment where all the technological components of the intervention, not only the Smart Glasses but also the telecommunication equipment and the energy source, had to be installed. All the staff involved had to be trained, even in the basic use of smartphones. Despite this, the new technology was readily accepted by the patients and the population, thanks to comprehensive community engagement by an NGO actor who has developed a long-term relationship with the community. It also was very attractive to the patients, as the health centre nurse could readily get advice in real-time and support from a hospital-based doctor, and decide together on the course of action.

The intervention was fully integrated into the formal public health system, and thus aimed at improving an important service for the population, and was perceived by the healthcare providers as enabling and empowering for them. Once fully functioning, the intervention facilitated their core tasks and improved working relations between the health centres and the referral hospital. In short, the intervention was perceived as improving their core tasks. Introducing telemedicine, especially in developing countries, frequently increases the workload of healthcare staff or doctors involved. However, in our project, none of the interviewees mentioned the additional workload of the intervention generated for mastering the technology and dealing with increased number of patients. This may be explained by the enthusiasm generated by learning a new technology and be part of an innovation which increases status. Additionally, more patients mean more patient fees, and increased staff income.

During the initial months of the new technology being available in the health centres, the number of visits increased sharply, probably mostly due to curiosity and initial enthusiasm, but the user rates remained significantly increased in the medium term.

The Smart Glasses and the moto-ambulances were most useful for difficult cases, in which the nurse was unsure whether to refer the patient to the hospital. In the context of a rural district like Kingandu, in DRC, quite often patients decide not to follow the referral advice and to seek alternative options in their immediate environment, which are plentiful amongst the private and traditional healthcare providers. It was a clear outcome from the project that referrals decided jointly between the health centre staff and a hospital-based doctor and facilitated by the moto-ambulance sent by the hospital, were willingly accepted by the patients, as they trusted that the hospital was expecting them and that it was the right place to take care of them.

So, the total number of consultations at health centres increased, but this did not result in an increase in the total number of referrals to the hospital. The field actors thought this was due to an increase in necessary referrals cancelled out by a decrease in unnecessary referrals, and increased trust by the health centre staff to manage cases locally; having the option to consult a doctor remotely at any moment for further advice.

The Smart Glasses element of the intervention also facilitated the introduction of new diagnostic technologies, such as the Hemocue, glucometer, and oximeter, through remote training and assistance, without the need for training workshops and consequent absence from the workplace.

The intervention was thus perceived as beneficial by all stakeholders involved. It also helped to use the resources of the health system more efficiently, which is a major gain given the extreme scarcity in which all actors have to operate in such a very low-resource setting.

It should be noted, however, that the introduction of such intervention required not only substantial investment in communication and energy systems but also substantial training, support, and troubleshooting, including for making rural staff acquainted with the basic operating skills of smartphones. This required a full-time dedicated staff member, qualified both as a medical doctor and as an IT expert, based in Kingandu and readily available for remote and on-site support, which was frequently needed over at least one full year. Even during the last four months of the project, when all equipment was fully functional and all staff fully trained, frequent troubleshooting remained necessary, but could increasingly be taken up by the district and hospital staff, who had become acquainted with the technology through their direct implication in the project.

Lessons learnt:

- Community engagement at all levels is critical when introducing a new technology in a sensitive area, such as healthcare.
- Applying a generic solution, such as telemedicine consultation, with a specific focus on maternal and neonatal healthcare, is not accepted in a first-line healthcare setting, but should be available for all aspects of the service package.
- The technical solution is an important and critical enabler, but it should be embedded in a larger solution to overcome other critical bottlenecks, such as in this case, emergency transport and improved diagnostics capacities.
- Providing additional ‘infrastructure’ to enable local healthcare workers to take appropriate action after diagnosis, is crucial (e.g. motorcycle for emergency referrals).
- Providing additional, more sophisticated ‘infrastructure’ can greatly enhance the quality of healthcare services delivered (e.g. diagnostic tools).
- The new technology can also greatly facilitate supervision and on-the-job training of health centre staff, to enhance up-skilling.
- Telemedicine should also be aligned with, or even trigger, income increases for the healthcare workers.
- Intense and broadly skilled project management should be available, covering the many diverse aspects such as community involvement, digital education of health centre staff, technical problem solving, and setting up of new processes in a medical context.
- Cost-wise: Initial investment cost is important, but operating cost, once installed, is acceptable under the condition that a financially sustainable alternative can be found for the expensive VSAT communication such as access to the internet/Wi-Fi, availability of (minimum) 3G, or affordable satellite communication. Indeed, in low-resource settings, the lowest level of technology that will do the job should be preferred to enable sustainability.

## Conclusion

Leveraging Smart Glasses for telemedicine in a remote rural district in the DRC can improve healthcare services. With extensive stakeholder engagement, investment in technology, moto-ambulances, and training, it was possible to increase the acceptability and use of the services, to improve diagnosis, treatment, and referral of patients to the hospital. Such intervention also creates opportunities for on-the-job training and supportive supervision. Under these conditions, a telemedicine intervention can improve the trust of the population in the health centres, empower the health centre staff, and create enthusiasm among all stakeholders

## Paper context: what is already known?

Delivery of primary healthcare in remote rural areas in sub-Saharan Africa faces many challenges, including among others acceptability by the users, low qualification and support of staff, difficult transport and communication.

Telemedicine leveraged by Smart Glasses as part of a comprehensive intervention (stakeholder engagement, introduction of new technology, and staff training) can contribute to overcoming these challenges.

Improved availability of internet access in rural Africa can support primary healthcare services and referrals.
